# Simultaneous Visualization of Microscopic Conductivity and Deformation in Conductive Elastomers

**DOI:** 10.1021/acsnano.3c10584

**Published:** 2024-01-15

**Authors:** Xiaobin Liang, Haonan Liu, So Fujinami, Makiko Ito, Ken Nakajima

**Affiliations:** † Department of Chemical Science and Engineering, School of Materials and Chemical Technology, Tokyo Institute of Technology, Ookayama 2-12-1, Meguro-ku, Tokyo 152-8550, Japan; ‡ Office of Society-Academia Collaboration for Innovation, Kyoto University, Gokasho, Uji, Kyoto 611-0011, Japan

**Keywords:** atomic force microscope, microscopic conductivity, conductive elastomers, flexible conductor, in situ

## Abstract

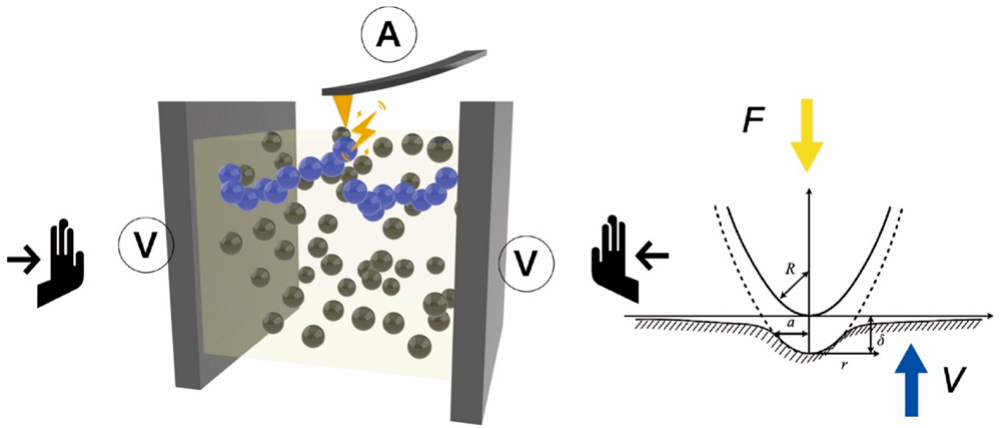

Conductive elastomers are promising for a wide range of applications in many fields due to their unique mechanical and electrical properties, and an understanding of the conductive mechanisms of such materials under deformation is crucial. However, revealing the microscopic conduction mechanism of conductive elastomers is a challenge. In this study, we developed a method that combines in situ deformation nanomechanical atomic force microscopy (AFM) and conductive AFM to successfully and simultaneously characterize the microscopic deformation and microscopic electrical conductivity of nanofiller composite conductive elastomers. With this approach, we visualized the conductive network structure of carbon black and carbon nanotube composite conductive elastomers at the nanoscale, tracked their microscopic response under different compressive strains, and revealed the correlation between microscopic and macroscopic electrical properties. This technique is important for understanding the conductive mechanism of conductive elastomers and improving the design of conductive elastomers.

Conductive elastomers, with their unique capability to combine deformability with excellent electrical conductivity, have been extensively applied in a variety of fields, including biosensors, flexible electronic devices, artificial muscles, and high-performance energy conversion materials.^1−5^ Traditional conductive elastomer designs utilize rigid conductors as base materials and achieve deformability through specific structural designs, particularly stretchable geometrical shapes.^6^ In recent years, research on inherently deformable conductors, such as conductive nanocomposites and quasi-solid ionic conductors, has gained attention as a revolutionary breakthrough in material technology.^7,8^ This innovative class of materials, offering broad application prospects, is accelerating progress in fields related to electronics, mechanics, and biology.^9−12^ To achieve both electrical conductivity and elasticity, conductive elastomers typically combine nanosized rigid conductors, such as carbon and metals with deformable elastomer polymers, or they incorporate conductive ions and conductive polymers into elastomer polymers.^13−18^ Over the past decade, researchers have devised a plethora of methods to fabricate stretchable conductive elastomers with impressive properties.^19−23^ However, to meet the diverse needs of practical applications, breakthroughs in issues such as the stability of conductivity, tunability of mechanical properties, and hysteresis of electrical performance are needed.^24−26^ Therefore, a thorough understanding of the mechanisms of electrical conductivity and mechanical performance of conductive elastomers is a key to improving stretchable conductive elastomers and is of critical importance to the efficient design and development of future conductive elastomers.

One of the challenges in elucidating the microscopic mechanisms of conductive elastomers lies in the accurate measurement and characterization of microscopic conductivity and deformation.^27,28^ Some researchers have attempted to elucidate the relationship between microscopic structures and macroscopic electrical conductivity through theoretical modeling and computer simulation.^29−33^ However, due to the complex conductive network structure of conductive elastomers (especially rigid conductor composite conductive elastic), it is very difficult to predict the change in this complex network structure when the material is deformed. Therefore, finding a method that can effectively characterize its deformation and conductivity at the micro/nanoscale has become a key issue in studying the microscopic mechanisms of conductive elastomers.

The atomic force microscopy (AFM) is a high-resolution scanning probe microscope that employs a nanoscale probe mounted on a flexible cantilever to scan the surface of a sample. By detecting the interactions between the probe and the surface, it characterizes a variety of properties such as mechanical, electrical, and chemical structures at the microscopic level.^34,35^ This technique is extensively used in research across various fields, including materials science, biology, and physics. AFM can measure the microscopic mechanical properties of a sample by pressing a nanoprobe against the material’s surface, which is known as the nanomechanical AFM technique.^36−39^ Furthermore, the conductive AFM (C-AFM) mode allows visualization of the microscopic electrical properties of the sample surface by forming a conductive loop between the probe and the sample.^40−42^ This feature of multiple functional characterizations gives AFM the potential to be an important tool for studying the microscopic mechanisms of conducting elastomers. In our recent work, we developed an approach based on nanomechanical AFM to successfully track the microscopic deformation and stress distribution of nanocomposite materials in situ.^43^ This approach will make it possible for us to simultaneously track the mechanical response and electrical response of a material during deformation at the nanoscale. In this work, we introduce a method combining in situ deformation, nanomechanical AFM and C-AFM, successfully characterizing the microscopic deformation and conductivity of conductive elastomer composites of carbon black (CB)/isoprene rubber (IR) and carbon nanotubes (CNTs)/hydrogenated nitrile rubber (HNBR). We performed microscopic conductive structure mapping to visualize the conductive network at the nanoscale. By tracking changes in the microscopic conductive structure and deformation, we revealed the correlation between the microscopic structure and macroscopic electrical properties. This innovative characterization method will provide the most intuitive and accurate information for the study of deformation and conductive mechanisms of conductive elastomers and is expected to make a great contribution to the evolution of fundamental theories and structural design.

## 1 Results and Discussion

### 1.1 Direct Visualization of Conductive Networks at the Nanoscale

**Figure 1:**
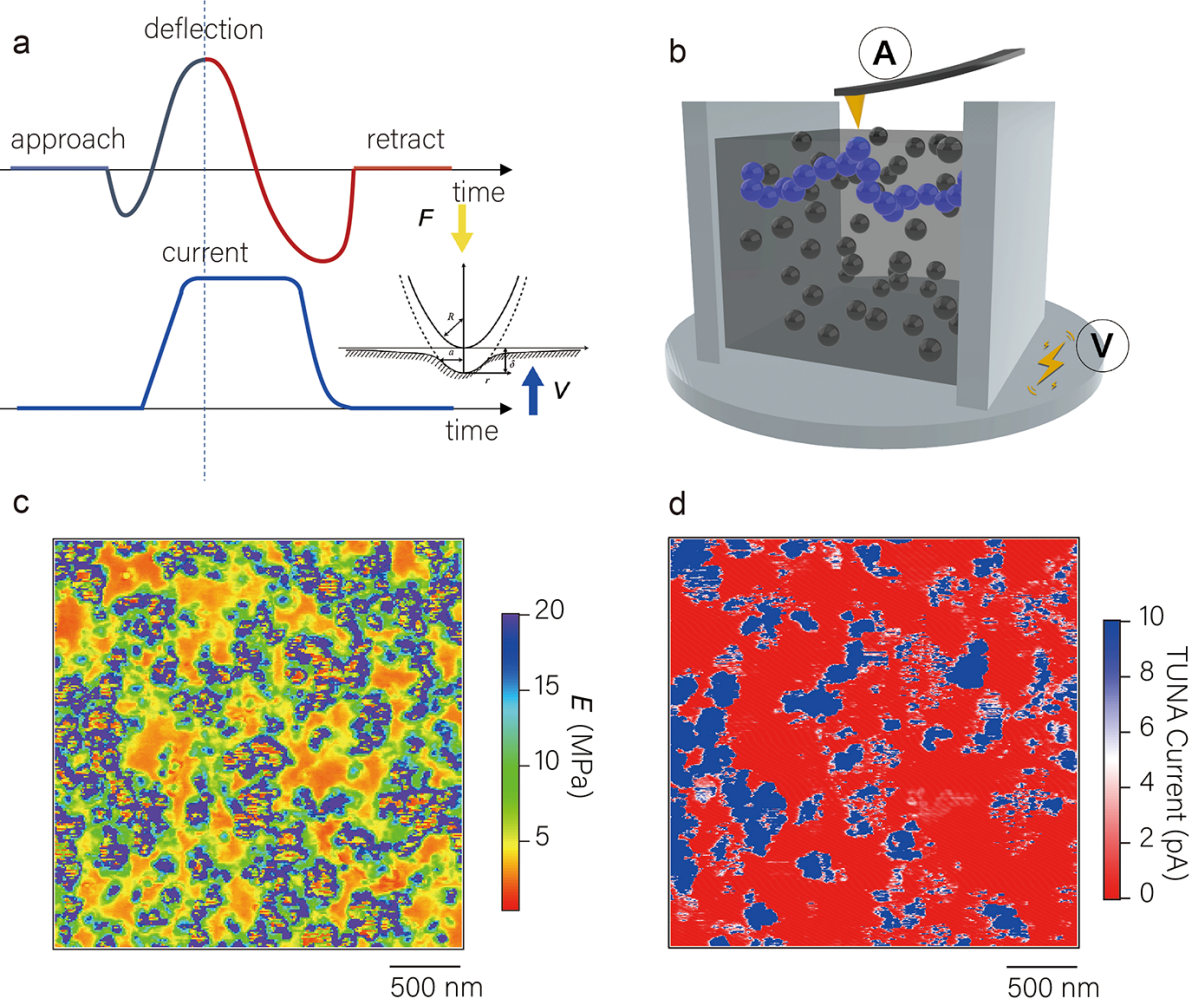
(a) Simultaneous measurement of the force curve and current information when the AFM probe contacts the surface. (b) Schematic diagram of the simultaneous measurement of the mechanical and electrical properties of CB/IR by AFM. (c) The nanoelastic modulus mapping of CB-28.6 wt %/IR in the undeformed state. (d) The nanocurrent mapping of CB-28.6 wt %/IR under undeformed conditions.

**Figure 2:**
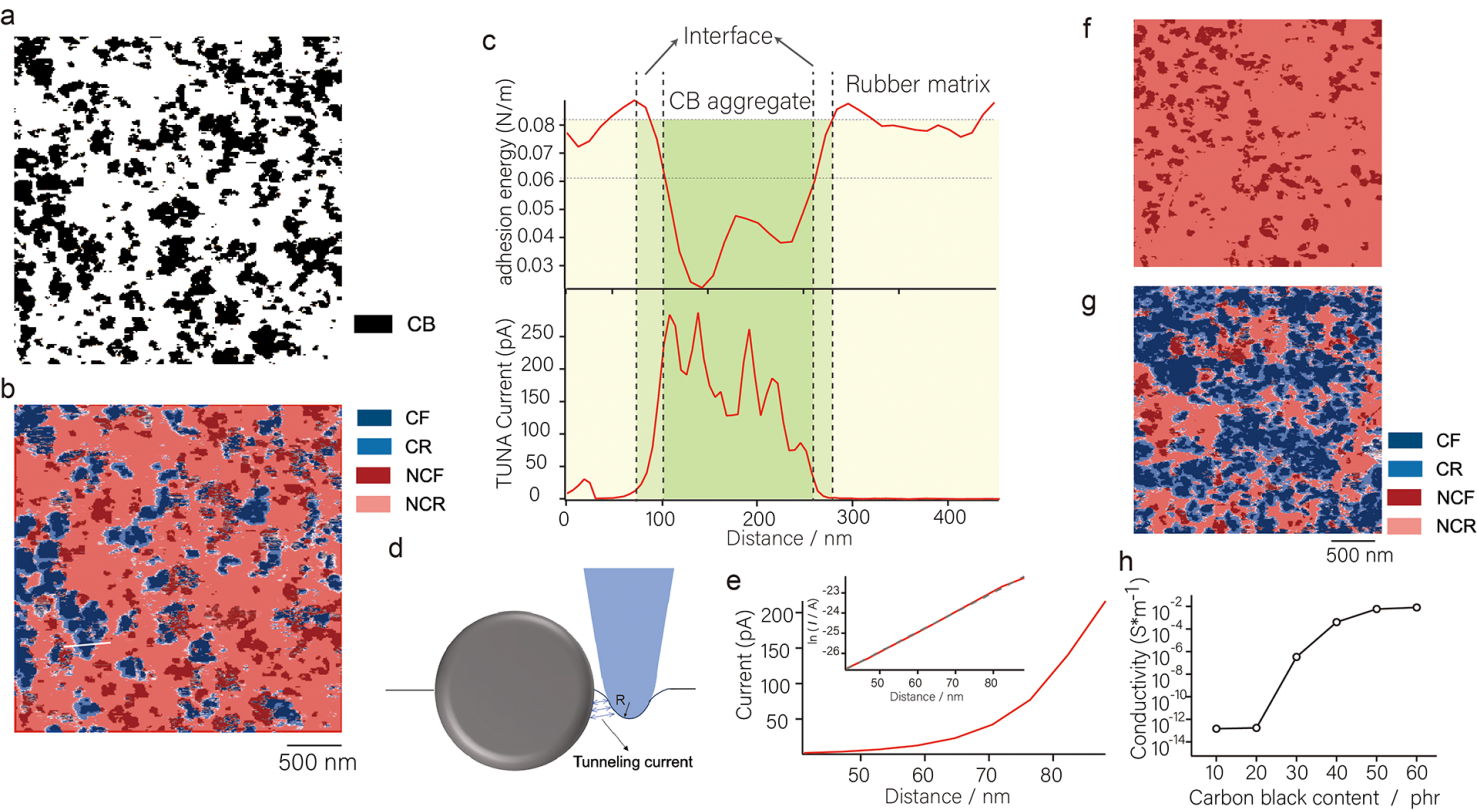
(a) Microscopic distribution images of CB particles and their aggregates in CB-28.6 wt %/IR in the undeformed state. (b) The microscopic conductive structure mapping of CB-28.6 wt %/IR is obtained by superimposing Figures 1d and 2d. (c) The cross-sectional profiles of the adhesion energy and current in the same region (white lines in Figure 2b). (d) Contact between the probe and the sample, which shows that the actual gap is smaller than the distance measured by AFM. (e) Correlation between the tunneling current and the distance between the AFM probe and CB. (f) The microscopic conductive structure mapping of CB-16.7 wt %/IR in the undeformed state. (g) The microscopic conductive structure mapping of CB-37.5 wt %/IR in the undeformed state. (h) The relationship between the macroscopic conductivity of CB/IR and the filling amount of CB.

In the study of microscopic conductive mechanisms in nanocomposite conductive elastomers, inferences about the correlation with macroscopic conductive properties are often drawn from observations of microscopic morphological changes in conductive fillers on the nanoscale. The lack of direct visualization at the microscale level hinders a deeper understanding of the conductive behaviors. AFM, an advanced instrument that characterizes both mechanical and electrical properties, is ideally suited to the needs of conductive elastomers. The nanoscale probe of the AFM can acquire mechanical characteristics by imprinting a small force onto the sample surface, while the simultaneous application of voltage can provide electrical properties of the sample, as illustrated in Figure 1a. Composite conductive elastomers are typically composed of conductive rigid fillers and nonconductive soft matrices that exhibit significant differences in their mechanical and electrical properties. These contrasting characteristics make it easier to characterize its microstructure by AFM. First, we measured the mechanical and electrical properties of the CB/IR system (shown in Figure 1b). Figure 1c,d show the nanoelastic modulus mapping and nanocurrent mapping of the 28.6% weight fraction CB nanoparticle composite IR (CB-28.6 wt %/IR), respectively. In the elastic modulus mapping, the blue region shows the high-modulus rigid CB particles or aggregates of CB particles and the orange region shows the low-modulus elastomeric matrix, with an interfacial region of intermediate-modulus material between the two. In the current mapping, there is a current flowing through the CB particle region (blue), which is due to the CB particles linking together to form a 3D conductive network. Importantly, not all CB particles have current flowing through them, which indicates the presence of CB particles or aggregated states of CB particles that are not connected to the network structure. Here, to clarify the distribution of CB particles, we used the multipeak fitting method to distinguish the CB, matrix and interface based on the difference in adhesion energy (please refer to the Supporting Information for details) and define the CB region in the AFM image, as shown in Figure 2a. Superimposing the distribution image of CB particles onto the current mapping yields a mapping, shown in Figure 2b, called microscopic conductive structure mapping. Excitingly, Figure 2b shows that the 4 types of regions are current-filled regions (CF), current-unfilled regions (CR), noncurrent-filled regions (NCF), and noncurrent-unfilled regions (NCR), with content fractions of *V*_CF_ = 13%, *V*_CR_ = 7%, *V*_NCF_ = 17%, and *V*_NCR_ = 63% (please refer to Table S1 for detailed data). It is clear that CF, NCF, and NCR correspond to the CB connected to the conductive network, the CB not connected to the conductive network, and the nonconductive elastomer matrix, respectively. This enables us to directly visualize the microscopic state of conductive particles connected to the conductive network has been directly visualized at the nanoscale and the connection rate of the conductive network of CB particles in CB-28.6 wt %/IR was *V*_CF_/(*V*_CF_ + *V*_NCF_) = 43.3%. Notably, the presence of CR regions was observed to be mostly distributed in the interfacial region. This region is considered to be derived from the conduction mechanism of the tunnel effect; that is, when two conductive particles are very close (a few nanometers), even if they are not in direct contact, electrons can complete conduction through the tunnel effect.^44,45^ The cross-sectional profiles of the adhesion energy and current in the same region (white lines in Figure 2b) are shown in Figure 2c, where the interfacial region, spanning approximately 20 nm in width, maintains its conductivity despite the rapid decline in current. The current at the interface originates from the tunneling current formed between the CB particles and the AFM probe; however, considering the deformation of the sample and the actual contact state (Figure 2d), the actual gap is smaller than the distance in Figure 2c. It is widely believed that the tunneling resistance increases exponentially with increasing gap, and the relationship between the tunnel conductivity σ and the gap *w* is as follows:^46^
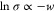
1

In Figure 2e, we show in detail the correlation between the tunneling current *I* and the distance of the AFM probe and CB. Strikingly, the ln(*I*) vs distance relationship shows a significant linear trend, which is consistent with theoretical predictions of tunneling resistance and gap rate. Further, we measured two samples with different volume fractions of CB, namely, CB-16.7 wt %/IR and CB-37.5 wt %/IR, and the results are shown in Figure 2f,g. Unsurprisingly, we did not observe any current in CB-16.7 wt %/IR, which is consistent with the measurement of its macroscopic conductivity (shown in Figure 2h). However, when the weight fraction of CB was increased to 37.5 wt %, it could be observed that more CB particles were connected to the conductive network, resulting in a significant increase in its conductive area, far exceeding conductive area of the CB-28.6 wt %/IR (Figure 2b). After calculation, the connection rate of CB particles in the conductive network reaches 79.4% in the CB 37.5 wt %/IR sample, which is almost twice that of the CB 28.6 wt %/IR sample. (Please refer to Table S2 for detailed data.) Still, the increased conductive pathways did not significantly increase the material’s macroscopic conductivity. This suggests that in CB conductive elastomers, the effect of tunnel resistance on its electrical conductivity may exceed the effect of the conductive network, which is consistent with some research results.^47^ Overall, our study provides an approach to directly visualize the microscopic conductive structure of conductive elastomers in real space, which provides crucial information for further revealing their conductive mechanism during deformation.

### 1.2 Microscopic Response of CB Conductive Network Structures during Deformation

**Figure 3:**
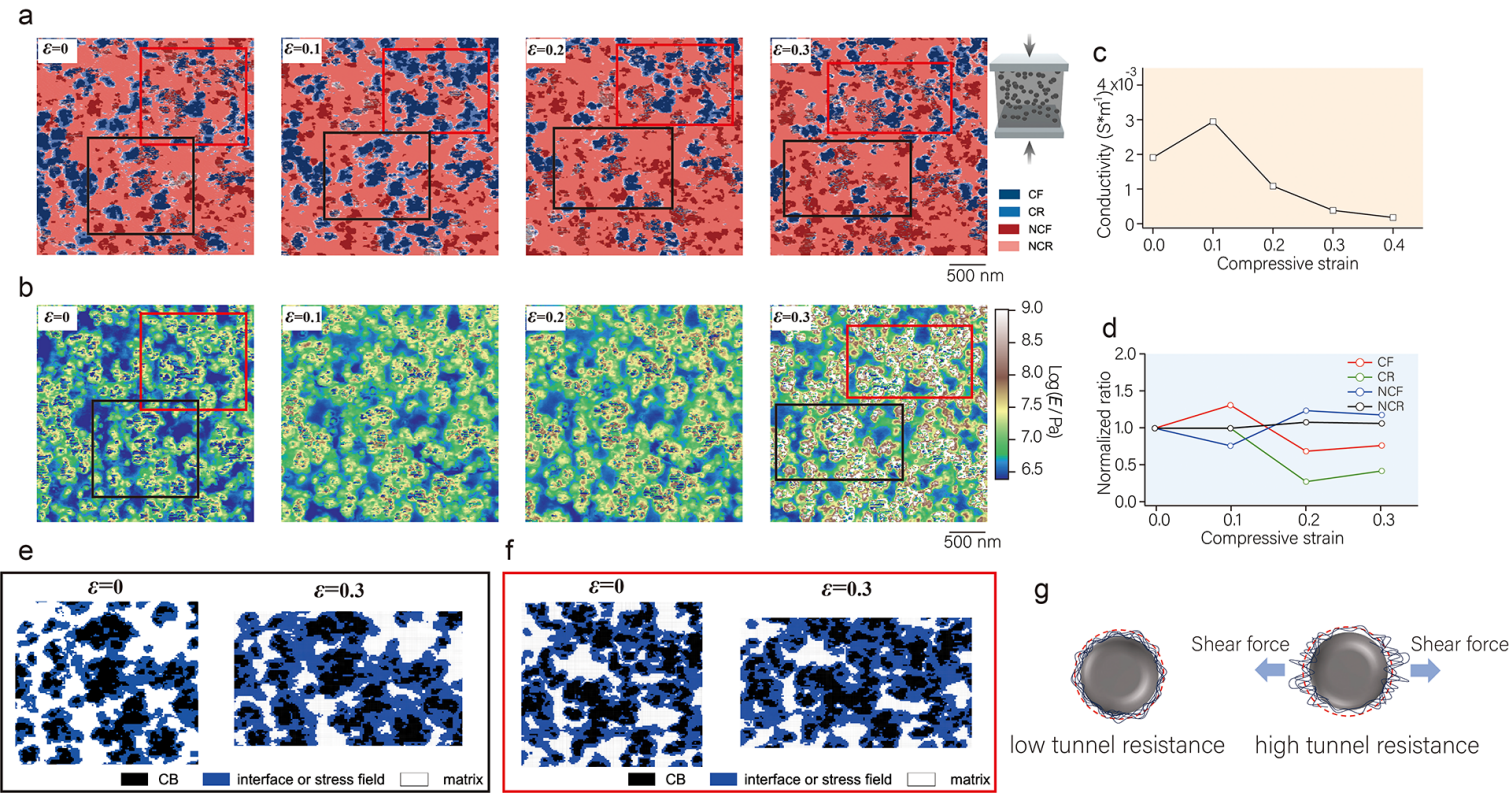
(a) Microscopic conductive structure mapping of CB-28.6 wt %/IR at compressive strains of ε = 0, 0.1, 0.2, and 0.3. (b) The elastic modulus mapping of CB-28.6 wt %/IR at compressive strains of ε = 0, 0.1, 0.2, and 0.3. (c) Macroscopic electrical conductivity of CB-28.6 wt %/IR as a function of compressive strain. (d) The normalized content ratios of CF, NCF, CR, and NCR in CB-28.6 wt %/IR as a function of compressive strain. (e) The stress network formation process in a specific region (black box in Figure 3a,b). (f) Stable CB network structure without significant stress network formation at strain (red box in Figure 3a,b). (g) The attached polymer segments on the CB surface detach from the surface under stress, resulting in a change in tunnel resistance.

For a long time, researchers have relied on inferring the relationship between macroscopic conductivity and microscopic morphology to understand the microscopic conduction mechanisms during deformation. The lack of a means to directly characterize microscopic features has seriously hindered our in-depth understanding of the evolution of conductivity during deformation. In a recent study, we proposed an AFM-based scheme for in situ tracking of the microscopic deformation of filled rubber during compression.^38^ Based on this method, we now propose a scheme: combining in situ deformation nanomechanical AFM with the C-AFM technique of the previous section. This integrated strategy will allow us to achieve in situ tracking of microscopic conducting structures during deformation, which is undoubtedly an exciting development that will provide us with a deeper understanding of the change in microscopic conductivity during deformation. Figure 3a,b shows the microscopic conductive structure mapping and elastic modulus mapping of the CB-28.6 wt %/IR samples in the same region under different compressive strain conditions, where the compressive stress is applied along the vertical direction. The stress bearing leads to a gradual increase in the elastic modulus mapping of the matrix region with increasing compressive strain, which is consistent with our previous reports. Notably, the microscopic conductive structure mapping shows that the CF region enlarges during the increase in compressive strain ε from 0 to 0.1; however, it starts to decrease significantly at the stage of the compressive strain ε increase from 0.1 to 0.3. This strain dependence of the CF content coincides with the trend in macroscopic conductivity, as shown in Figure 3c. This phenomenon has been reported in some earlier studies and has been referred to as the negative-pressure coefficient effect.^47^ The proximity of CB particles to each other under compressive stress leads either to an increase in direct contact or to a decrease in the thickness of the CB spacer, which creates more tunneling currents and thus increases the number of conductive paths. This increase in network connections causes an increase in the macroscopic conductivity. However, when the compressive stress continues to increase, the large deformations induced lead to a large amount of damage to the conductive network when the probability of network failure is greater than the probability of reconnection formation. Therefore, at large strains, the conductivity decreases with an increasing strain, a phenomenon known as the positive-pressure coefficient effect. This visualization technique provides an important perspective for understanding the conduction mechanism of CB/IR conducting elastomers in stress deformation.

To gain insight into the origins of this phenomenon, we quantitatively analyzed the ratios of CF, NCF, CR, and NCR as a function of strain. Figure 3d shows the normalized content of the four components as a function of strain. When the compressive strain was 0.1, the content of CF increased to 1.38, while that of NCF decreased to 0.76, which indicated that the network connection rate of CB increased. However, when the compressive strain increased to 0.2, the content of CF decreased to 0.71, while the content of NCF increased to 1.23, at which point the conductive network was heavily disrupted. This interesting reversal phenomenon reveals a close relationship between network connectivity and stress (detailed data are given in Table S1). Furthermore, we tracked the changes in microscopic stress and microscopic current in specific regions. In a previous study, we identified a stress transfer mechanism: at low strain, the stress is mainly distributed in the area around the CB, and the stress concentration area expands and connects with the increase in strain, forming a stable stress network structure to bear the stress. Figure 3e shows the stress network formation process in a specific region (black box in Figure 3b). Since this process is accompanied by a large change in the displacement of the CB particles, we can see that the conductive network in this region clearly connects and breaks with strain (see black box in Figure 3a, and please refer to the Supporting Information for a detailed discussion of the stress network). In contrast, the region in Figure 3f (red box in Figure 3b) forms a stable CB network structure when it is not deformed, is difficult to deform under strain, and the conductive network in this region also maintains its stability when strained. We consider that the formation of the stress network is an important cause of the change in the conductive network. In addition, we note that the thickness of the CR region, which represents the tunneling current between the probe and the CB, decreases significantly with increasing strain. This phenomenon may be attributed to the fact that during deformation the stress is more concentrated at the interface, resulting in the polymer chain segments attached to the surface of the CB partially detaching from the CB in response to the stress (see Figure 3h). This structural change leads to an increase in the tunnel barrier height and a decrease in the tunnel conduction distance, which further affects the microscopic conductivity. These findings contribute to our deeper understanding of the evolution of the conductive network structure under stress.

**Figure 4:**
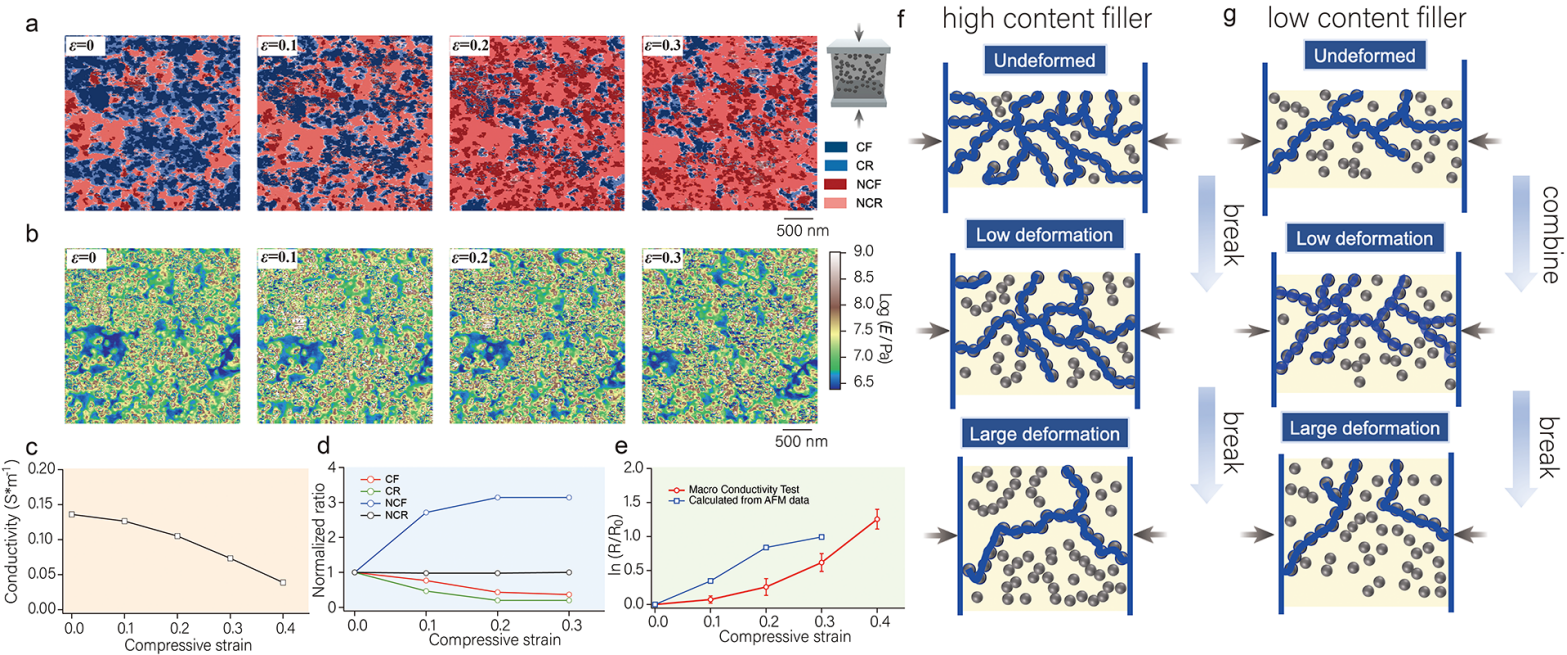
(a) Microscopic conductive structure mapping of CB-37.5 wt %/IR at compressive strains of ε = 0, 0.1, 0.2, and 0.3. (b) The elastic modulus mapping of CB-37.5 wt %/IR at compressive strains of ε = 0, 0.1, 0.2, and 0.3. (c) Macroscopic electrical conductivity of CB-37.5 wt %/IR as a function of compressive strain. (d) The normalized content ratios of CF, NCF, CR, and NCR in CB-37.5 wt %/IR as a function of compressive strain. (e) The relationship between resistance and strain for CB-37.5 wt %/IR: the red line is the result of the macroscopic conductivity measurement, and the blue line is the calculation result of adding  obtained by AFM into eq 6. (f) Schematic diagram of the relationship between the conductive network and the strain in a high-CB-content elastomer. (g) Schematic diagram of the relationship between the conductive network and the strain in a low-CB-content elastomer.

Next, we investigated the microscopic conductive structure mapping and elastic modulus mapping of CB-37.5 wt %/IR, as shown in Figure 4a,b. Unlike the case of CB-28.6 wt %/IR, CB-37.5 wt %/IR does not exhibit a negative coefficient stress at the early stage of deformation, and the CF content shows a monotonically decreasing trend with increasing strain. Compared with CB-28.6 wt %, the CB network connection rate in CB-37.5 wt %/IR is as high as 79.4%, indicating that most of the CB connects to form a stable network structure. The CB/IR sample with a high filling content forms a complete CB spatial network structure when it is not deformed. Therefore, the CB network bears the stress at the initial stage of the strain, and the change in the displacement of the CB leads to a large amount of damage to the network. In this process, the network reconnection efficiency was far less than the damage to the network. The normalized content of the four components as a function of strain (see Figure 4d) shows that the CF content decreases to 0.27 and the NCF increases to 2.9 at strains from 0 to 0.2. Notably, this trend becomes slower at strains from 0.2 to 0.3, which may be attributed to the fact that the connectivity of the CB network is already very low and the rate of network damage decreases, making the contribution of network reorganization relatively increased (the detailed original data are shown in Table S2). The macroscopic conductivity shows the same trend as that in the microscopic results, as shown in Figure 4c. The macroscopic resistance grows exponentially with strain, and several studies have attempted to create models to predict the relationship between resistance and strain.^48,49^ One of the classic models describing the relationship between resistance *R* and mean tunnel distance *d* is based on the Simons function, expressed as follows:^50^
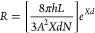
2
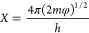
3where *N* is the number of conducting paths and *L* is the number of particles within a conducting path; *e*, *m*, and *h* are the electron charge, mass, and the Plank constant, respectively; *A*^2^ is the effective area; and φ is the barrier height between adjacent nanosheets. When a small strain is applied (the conducting path is not disrupted), the change in resistance can be described by the original tunneling distance *d*_0_ and the strain ε as
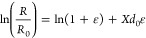
4When the strain is large, the change in *N* conforms to the tunnel failure model, which can be described as
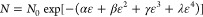
5Based on the tunneling and destruction model, the relationship between resistance and strain can be obtained as follows:
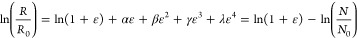
6In previous studies, the conductive path *N* has always been regarded as a parameter that is difficult to characterize directly or quantitatively through experiments. However, by employing AFM experiments, we are able to directly measure the volume proportionality between *V*_CF_ and *V*_NCF_, where an approximate correspondence between *V*_CF_ and *N* exists. Based on this relationship, we can further conclude that *N*/*N*_0_ ≈ *V*_CF_/*V*_CF__0_, i.e., . Substituting the  obtained from the AFM experiment into eq 6, the calculated relationship between resistance and strain is shown in Figure 4e. The AFM calculation results are generally consistent with the macroscopic resistance strain data, but there are still deviations. The reason for this deviation may be that the conductive network structure composed of CB particles is more prone to rupture under strain. In addition, the role of the tunneling effect is neglected in the model, especially at the interface where CB/IR has a strong interaction, and the effect of the tunneling effect is more obvious. We need to revisit the model of the conductive strain theory in the future. In short, we find that the conductive network is closely related to the stress network mechanism. At high filling levels, the CB network structure bears the stress, and the strain leads to network disruption of the CB and thus to the reduction of the conductive path, as shown in Figure 4h. On the other hand, at low CB filling levels, the stress network structure mainly bears the stress, and the nearby CB particles are more prone to displacement, which in turn leads to the connection and disruption of the conductive network, as shown in Figure 4g.

### 1.3 Microscopic Response of CNT Conductive Network Structure during Deformation

**Figure 5:**
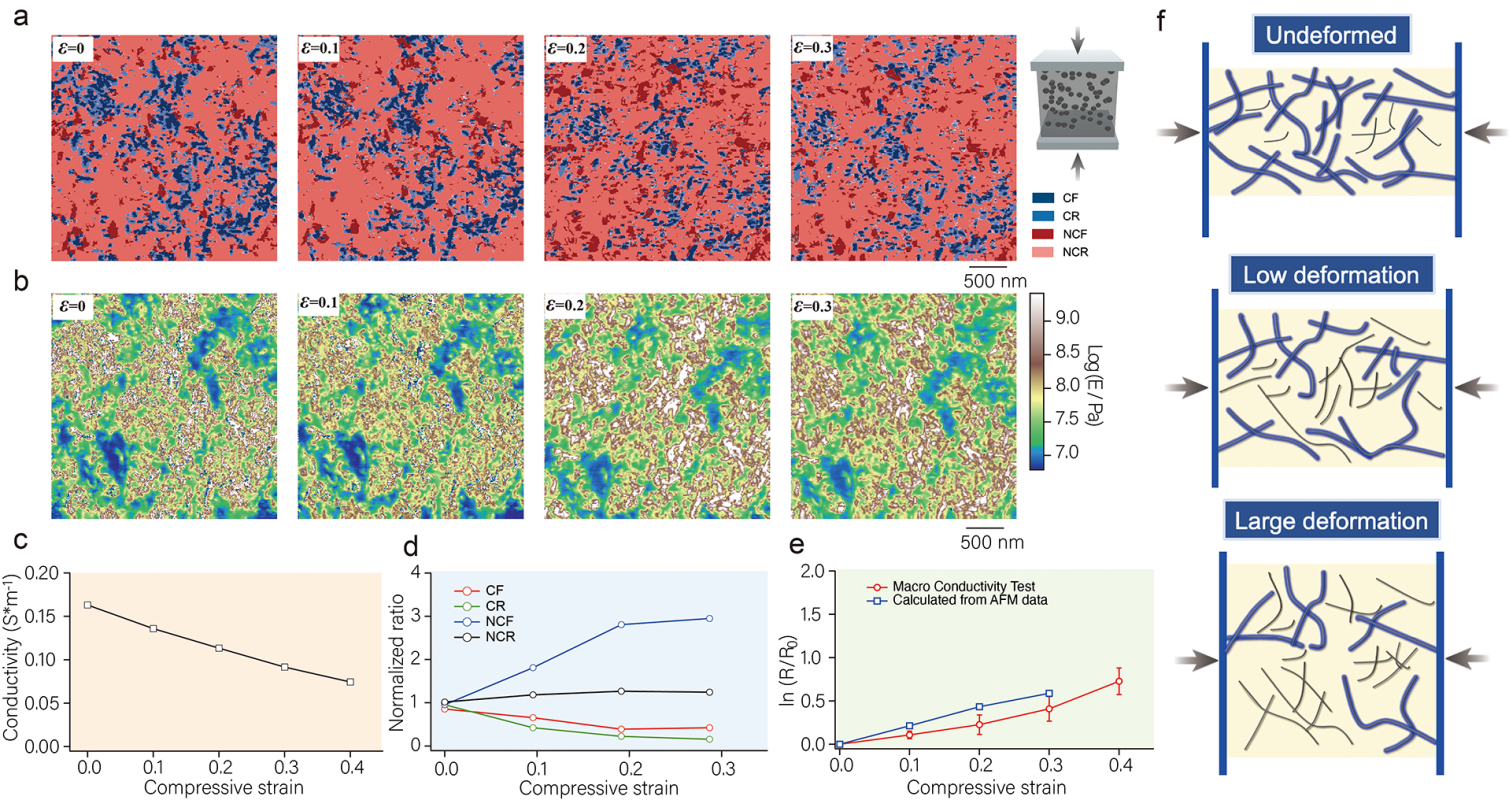
(a) Microscopic conductive structure mapping of CNT-9.1 wt %/HNBR at compressive strains of ε = 0, 0.1, 0.2, and 0.3. (b) The elastic modulus mapping of CNT-9.1 wt %/HNBR at compressive strains of ε = 0, 0.1, 0.2, and 0.3. (c) Macroscopic electrical conductivity of CNT-9.1 wt %/HNBR as a function of compressive strain. (d) The normalized content ratios of CF, NCF, CR, and NCR in CNT-9.1 wt %/HNBR as a function of compressive strain. (e) The relationship between resistance and strain for CNT-9.1 wt %/HNBR: the red line is the result of the macroscopic conductivity measurement, and the blue line is the calculation result of adding  obtained by AFM into eq 6. (f) Schematic diagram of the relationship between the conductive network and the strain in the CNT-filled elastomer.

Among stretchable conductive elastomers, CB composite elastomers suffer from low electrical conductivity and poor stability, which limit their application range. One-dimensional (1D) fillers such as CNTs and metal nanowires have attracted much attention due to their ability to maintain sufficient electrical conductivity under large deformations.^51^ Here, we choose CNT-9.1 wt %/HNBR as the experimental object to track the strain response of the conductive network structure in the 1D conductive elastomer. Figure 5a and Figure 5b show the microscopic conductive structure mapping and elastic modulus mapping of CNT/HNBR under different compressive strains. Similar to that of CB/IR, the microscopic conductive structure mapping of CNT/HNBR also shows four different components: CF, NCF, CR, and NCR. Although the content of CNTs is only 9.1 wt %, the unique 1D structure results in a very low percolation threshold relative to CB. Surprisingly, the conductive network connection rate of CNT-9.1 wt %/HNBR is as high as 75.0%, which is much higher than the 43.5% of CB-28.6 wt %/IR. The high network connection rate is the reason for the low percolation threshold of CNTs. In the microscopic conductive structure mapping of CNT-9.1 wt %/HNBR, it can be seen that as the compressive strain increases, the ratio of conductive components in CNT/HNBR shows an overall downward trend, which is consistent with the relationship between the macroscopic conductivity and strain (Figure 5c), but there are no obvious network reorganization and destruction processes.

The results of the quantitative analysis of the components show a similar trend (Figure 5d). At a strain of 0.3, the normalized content of the CF region decreases to only 0.78, a value far exceeding the 0.18 of CB-37.5 wt %/IR, which indicates that the CNT conductive network is not damaged significantly during the strain process and that the CNTs with high aspect ratios form a more stable network structure. Notably, the decreased ratio of CR with strain is much greater than that of CF, which decreases to 0.37 at a strain of 0.3, and the interface of the CNT/HNBR is affected by stress, resulting in an increase in tunneling resistance (detailed data in Table S3). The tunneling conductivity effect of both the CB system and CNT system has a significant decrease, but the macroscopic conductivity of the CNT system does not show a significant decrease, which is because the conductive network mainly consists of the CNTs in direct contact with other CNTs and thus is not dominated by tunnel resistance. Substituting the AFM-measured  of the CNTs into eq 6 in the previous section, a resistance vs strain relationship is obtained, as shown in Figure 5e. We can see that the calculated values for the CNT system are closer to the macroscopic data, relative to the CB/IR system. This suggests that the 1D filler system is more consistent with the model’s description of the conductive path-strain relationship. Figure 5f shows a schematic diagram of the change in the CNT conductive elastomer with strain in which the conductive path of the CNT decreases slowly with the strain. CNT and CB show different microscopic conductive mechanisms due to the difference in shape and aggregation state. In conclusion, this simultaneous visualization of microscopic conductivity and deformation is expected to help us uncover the microscopic mechanism of conductive elasticity.

## 2 Conclusions

We propose an approach based on the combination of in situ deformation nanomechanical AFM and C-AFM to directly visualize the microscopic conductive network of conductive elastomers at the nanoscale and their microscopic responses under strain. This method provides a microscopic mapping of the conductive structure, which allows us to directly identify whether the conductor packing is connected to a conductive network or not in real space and to calculate the network connectivity of the conductor and track its change with strain. In addition, we observe the presence of a tunneling conductive region close to the conductor by this method.

We measured conductive elastomer systems containing CB fillers and CNT fillers and found that the conductive network is closely related to the stress network mechanism. In materials with a low CB filling content, stress transmission is mainly realized through the stress network structure, and the CB particles near the stress network structure are more likely to be displaced. This displacement may promote the connection and destruction of the conductive network, thereby affecting the overall conductive performance. In contrast, in materials with a high CB filling content, the CB forms a network structure and plays a role in bearing stress. When the material is strained, this CB network is damaged, reducing the efficiency of the entire conductive network. In contrast, the network structure in the 1D structured CNT system is more stable and its strain causes less damage to the conductive network. In addition, we observed that the tunnel conduction area gradually decreases with strain, which may be due to the stress-induced effect of the polymer chain segments detached from the filler. Finally, by verifying the relationship between the network connectivity of conductive fillers and the macroscopic conductivity, we found that the 1D filler system is more in line with the classic model’s description of the relationship between the conductive path and the strain. The AFM force mode-electric mode combination method provides important information for an in-depth study of the conduction mechanism of conductive elastomers and fills the gap in the characterization of microscopic conductivity during deformation.

In conclusion, this approach enables the direct visualization of microscale conductive structures and tracking of their evolution during deformation, thereby facilitating a deeper understanding of the microscopic mechanisms underpinning the conductivity and mechanical properties of conductive elastomers. More importantly, this method is universally applicable to a wide range of conductive elastomer materials, including nanocomposites, conductive polymer gels, and ionic conductors, making it an indispensable tool in the field of conductive elastomer research.

## 3 Methods

### 3.1 Materials

The materials used in this study are IR rubber compounded with high-wear-resistance furnace-grade CB (N330) and H-NBR rubber compounded with multiwall carbon nanotubes (MWCNTs, C7000). Detailed formulations of these materials are listed in Table S4. These materials are commercially available.

To obtain a smooth surface suitable for AFM imaging, we used a Leica EM FC6 (Leica Microsystems GmbH Wetzlar, Germany) to perform ultrathin sectioning of the sample at −120 °C, with the cutting direction perpendicular to the compression direction. Afterward, we performed AFM measurements using a specially designed sample holder for controlled compression.

### 3.2 Characterization

AFM measurements were performed using Nanoscope V and MultiMode 8 in PeakForce TUNA (PFTUNA) mode (Bruker AXS, U.S.A.). The PFTUNA mode combines the PeakForce QNM mode with conductivity (TUNA) measurements, making it possible to image mechanical and electrical properties in parallel at the nanoscale. During a measurement cycle, the probe approaches and leaves the sample surface, generating a force curve. The maximum current value is also collected when the probe is in contact with the sample, thereby obtaining a microscopic current image of the sample surface. For the calculation of the micromechanical properties, we extracted the force curves from the raw data and analyzed the force curves using the Johnson–Kendall–Roberts (JKR) contact model. Detailed calculations are given in the Supporting Information.

Here, the sample was scanned with a peak tapping force of approximately 4 nN by using a cantilever beam (PF-TUNA, Bruker, U.S.A.) with a nominal spring constant of 0.4 N/m. The tip radius *R* of the probe was determined to be 15 nm by a Nioprobe TipCheck sample (Aurora NanoDevices Inc., Canada). The actual spring constant was measured by a thermal tuning method. The Z piezo has an oscillation frequency of 1.0 kHz and a peak force amplitude of 250 nm. The scan rate was 0.5 Hz. Force curves were collected at a resolution of 256 pixels × 256 pixels on a selected 3.0 μm surface area.

The sample was set in a metal sample holder, a voltage of 3 V was applied, and the front and back of the cantilever were coated with platinum–iridium to provide a current return path. Notably, the selection of the peak tapping force is very important, with the appropriate magnitude of force satisfying only the small deformation to improve resolution but also to maintain some contact to obtain stable current information.

For electrical property characterization, the conductivity of 2 mm thick undeformed samples was measured by using a high-resistance instrument (Hiresta-UX MCP-HT800, Mitsubishi, Japan). The conductivity of CB/IR and CNT/H-NBR under different compressive strains was measured by a homemade two-probe method using a multimeter (GDM-8341, GW Instek, China).

## Supporting Information Available

A schematic of force–deformation curve; Binarized image of the CB and non-CB regions; Ternary images based on separation at compressive strains ε = 0, 0.1, 0.2, and 0.3; The content ratio of CF, NCF, CR, and NCR at compressive strain ε = 0, 0.1, 0.2, and 0.3; Formulation of the CB-filled isoprene rubber and CNT-filled/HNBR (PDF)
