# Supplementary material for: Simultaneous Visualization of Microscopic Conductivity and Deformation in Conductive Elastomers
Source: ACS Nano. 2024 Jan 15;18(4):3438–46. doi: 10.1021/acsnano.3c10584 (PMC10832062; doi:10.1021/acsnano.3c10584)
Supplement: Supplementary file 1 — nn3c10584_si_001.pdf [file nn3c10584_si_001.pdf]

Supporting Information

Simultaneous Visualization of Microscopic Conductivity and Deformation in Conductive Elastomers

Xiaobin Liang [1*], *Haonan Liu* [1], *So Fujinami* [2], *Makiko Ito* [1], *Ken Nakajima* [1*].

[1]Department of Chemical Science and Engineering, School of Materials and Chemical Technology, Tokyo Institute of Technology, Ookayama 2-12-1, Meguro-ku, Tokyo 152-8550, Japan

[2]Office of Society-Academia Collaboration for Innovation, Kyoto University, Gokasho, Uji, Kyoto 611-0011, Japan

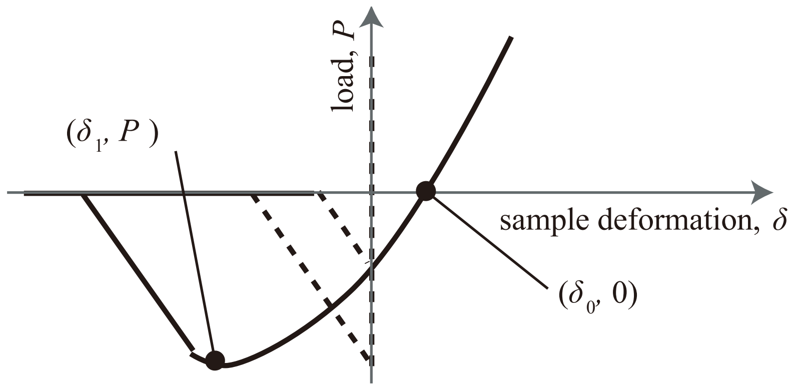

Figure S1. A schematic of force–deformation curve.

The contact of the AFM probe with the sample surface can obtain a force curve, as shown in Figure S1. The force curves were analyzed using the Johnson–Kendall–Roberts (JKR) contact model.[1] The Young's modulus E and adhesion energy w were represented by the following two equations based on the JKR model:

$$E = \frac{3(1-v^2)}{4}\frac{-1.27P}{\sqrt{R(\delta_0-\delta_1)^3}} \quad (1)$$

$$w = -\frac{2P}{3\pi R} \quad (2)$$

where v is Poisson's ratio, R is the radius of curvature for the probe tip, δ is the sample deformation, and P (< 0) is the maximum adhesive force.[2]

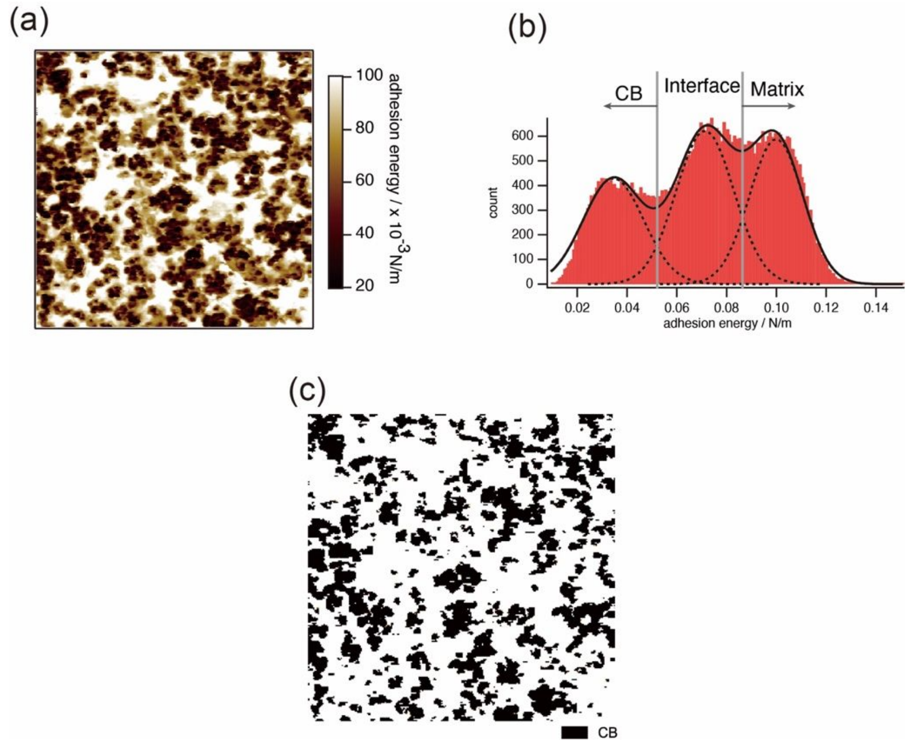

Figure S2. (a) The nano-adhesion energy mapping of CB-28.6 wt%/IR at the undeformed (the scan size is 3.0 μm). (b) Histogram of the adhesion energy. (c) Binarized image based on multi-peak fitting.

To further confirm the distribution of CB particles, it is necessary to distinguish the phases in the AFM images. Figure S2a shows nano-adhesion energy mapping of CB-28.6 wt%/IR at the undeformed, whose histogram (Figure S2b) is well described by three independent Gaussian functions, making it possible to distinguish CB, interface, and rubber matrix in the image. A binarized image of the CB and non-CB regions can be obtained by separating the phases as shown in Figure S2c.

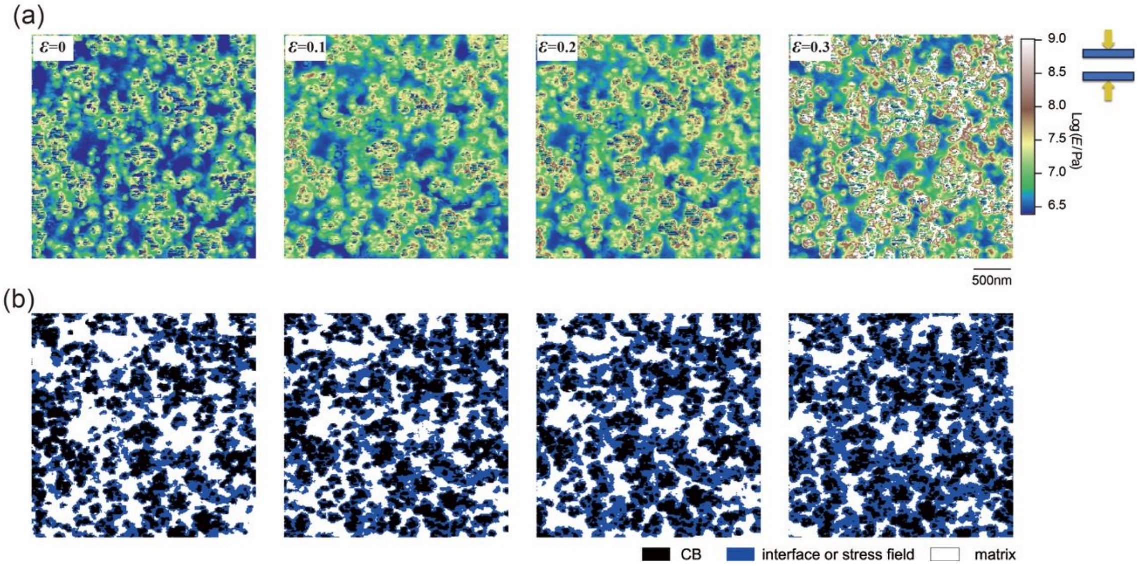

Figure S3. (a) The elastic modulus mapping of CB-28.6 wt%/IR at compressive strains of $\varepsilon = 0$, 0.1, 0.2 and 0.3. (b) Ternary images based on separation at compressive strains $\varepsilon = 0$, 0.1, 0.2, and 0.3.

Figure S3a shows a logarithmic image of the elastic modulus of CB-28.6 wt%/IR when undeformed. Using the separation method shown in Fig. S2 made it possible to evaluate the phases. The three components are the low-modulus IR matrix, the high-modulus CB phase, and the intermediate modulus region. It is important to note that for undeformed samples, the intermediate modulus region is considered to be the interface, whereas, for deformed samples, the intermediate modulus region contains the interface and the stressed rubber matrix near the interface. By separating each phase, the ternary images of the CB-28.6 wt%/IR were obtained, as shown in Fig. S3b. We found that the proportion of the stressed rubber matrix (blue region) gradually increases with the increase of strain and is connected to each other to form a network structure called a "stress network". Please refer to our previous report[3] for a detailed discussion of the formation of this stress network.

Table S1. The content ratio of CF, NCF, CR, and NCR in CB-28.6 wt%/IR at compressive strain ε = 0, 0.1, 0.2 and 0.3, respectively.

Strain	0	0.1	0.2	0.3
$\boldsymbol{V_{CF-CB28.6wt\%}}$	13%	17%	9%	10%
$\boldsymbol{V_{CR-CB28.6wt\%}}$	7%	7%	2%	3%
$\boldsymbol{V_{NCF-CB28.6wt\%}}$	17%	13%	21%	20%
$\boldsymbol{V_{NCR-CB28.6wt\%}}$	63%	63%	68%	67%

Table S2. The content ratio of CF, NCF, CR, and NCR in CB-37.5 wt%/IR at compressive strain ε = 0, 0.1, 0.2 and 0.3, respectively.

Strain	0	0.1	0.2	0.3
$\boldsymbol{V_{CF-CB37.5wt\%}}$	27%	21%	13%	14%
$\boldsymbol{V_{CR-CB37.5wt\%}}$	15%	7%	4%	3%
$\boldsymbol{V_{NCF-CB37.5wt\%}}$	7%	13%	20%	21%
$\boldsymbol{V_{NCR-CB37.5wt\%}}$	51%	59%	63%	62%

Table S3. The content ratio of CF, NCF, CR, and NCR in CNT-9.1 wt%/HNBR at compressive strain ε = 0, 0.1, 0.2 and 0.3, respectively.

Strain	0	0.1	0.2	0.3
$\boldsymbol{V_{CF-CNT9.1wt\%}}$	18%	16%	14%	13%
$\boldsymbol{V_{CR-CNT9.1wt\%}}$	8%	4%	4%	3%
$\boldsymbol{V_{NCF-CNT9.1wt\%}}$	6%	8%	15%	15%
$\boldsymbol{V_{NCR-CNT9.1wt\%}}$	68%	72%	68%	69%

Table S4. Formulation of the CB-filled IR and CNT-filled HNBR.

**Component**	**CB-16.7 wt%/IR**	**CB-28.6 wt%/IR**	**CB-37.5 wt%/IR**	**CNT-9.1 wt%/HNBR**
**IR**	100phr	100 phr	100 phr	-
**H-NBR**	-	-	-	100 phr
**Sulfur**	2.0 phr	2.0 phr	2.0 phr	-
**Stearic acid**	1.0 phr	1.0 phr	1.0 phr	-
**Zinc oxide**	5.0 phr	5.0 phr	5.0 phr	3.0 phr
**Cyclohexylbenzothiazole-2-sulfenamide**	1.0 phr	1.0 phr	1.0 phr	-
**Peroximone**	-	-	-	8.0
**CB**	20 phr	40 phr	20 phr	
**CNT**				10phr

phr: per hundred rubber

References

1. Johnson, K. L.; Kendall, K.; Roberts, A. D.; Surface Energy and the Contact of Elastic Solids, *Proc. Roy. Soc. Lond. A* **1971**, *324*, 301.
2. Sun, Y.; Akhremitchev, B.; Walke, G. C. Using the Adhesive Interaction between Atomic Force Microscopy Tips and Polymer Surfaces to Measure the Elastic Modulus of Compliant Samples. *Langmuir* **2004**, *20*, 5837.
3. Liang, X.; Kojima T.; Ito, M.; Amino, N.; Liu, H.; Koishi, M.; Nakajima, K. In Situ Nanostress Visualization Method to Reveal the Micromechanical Mechanism of Nanocomposites by Atomic Force Microscopy, *ACS Appl. Mater. Interfaces* **2023**, *15*, 12414.
